# Supplementary material for: Synovial Macrophages Expression of OX40L Is Required for Follicular Helper T Cells Differentiation in the Joint Microenvironment
Source: Cells. 2022 Oct 21;11(20):3326. doi: 10.3390/cells11203326 (PMC9601235; doi:10.3390/cells11203326)
Supplement: Supplementary file 1 [file cells-11-03326-s001.zip › cells-1950907-supplementary.pdf]

Supplementary table

Table S1. Clinical information of the participants (mean ± SD).

Participants, n=35	Osteoarthritis=19	Rheumatoid arthritis=16	p value
Age (yeas)	54.16 ± 6.212	53.13 ± 7.051	0.648
Weight (kg)	62.58 ± 6.203	65.06 ± 5.859	0.235
BMI (kg/m^2)	24.68 ± 0.938	24.34 ± 0.726	0.244
Smoking status (Yes/No)	1/18	1/15	0.815
Drinking status (Yes/No)	2/17	2/14	0.721
Diabetes type II (Yes/No)	4/15	3/13	0.976
Hypertension (Yes/No)	3/16	4/12	0.755
Number of medications	1±0.21	0.95±0.19	0.659
Sex (Male/Female)	11/8	3/13	/
Disease duration (month)	13	18	/
DAS28 scores	/	4.5±0.3	/

Supplementary figures and legends

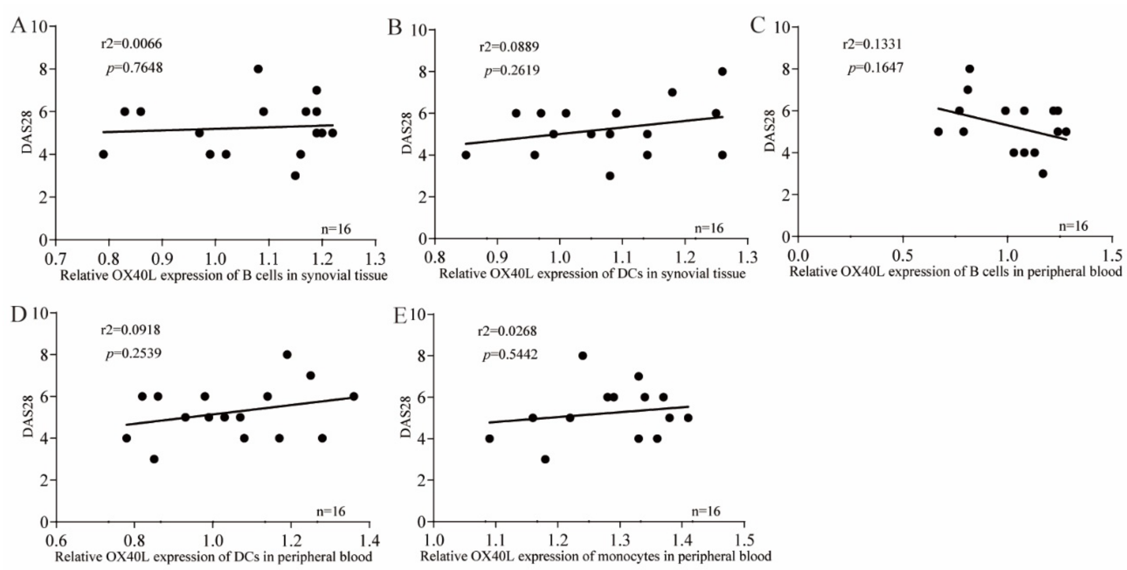

Figure S1. Correlation analysis between B cells or dendritic cells and DAS28. (A)

Correlation analysis of OX40L level of CD19^{+} B cells in the synovial tissues with DAS28 in RA patients. (B) Correlation analysis of OX40L level of CD11b^{+}CD11c^{+} DCs in the synovial tissues with DAS28 in RA patients. (C) Correlation analysis of OX40L level of CD19^{+} B cells in the peripheral blood with DAS28 in RA patients. (D) Correlation analysis of OX40L level of CD11c^{+} DCs in the peripheral blood with DAS28 in RA patients. (E) Correlation analysis of OX40L level of CD14^{+} monocytes in the peripheral blood with DAS28 in RA patients. *P*<0.05 represents that the vertical coordinate is correlated with the horizontal coordinate, otherwise there is no correlation (Linear regression was used).

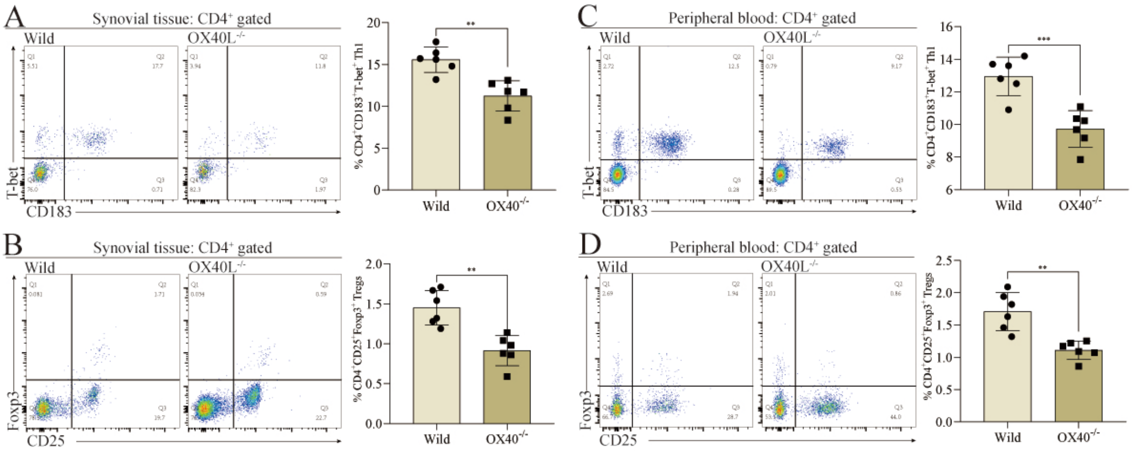

Figure S2. **The proportion of Th1 or Tregs in CD4^{+} T cells**. (A) The proportion of CD183^{+}T-bet^{+} Th1 in CD4^{+} T cells in the synovial tissues of wild and OX40L$^{-/-}$ mice. (B) The proportion of CD25^{+}Foxp3^{+} Tregs in CD4^{+} T cells in the synovial tissues of wild and OX40L$^{-/-}$ mice. (C) The proportion of CD183^{+}T-bet^{+} Th1 in CD4^{+} T cells in the peripheral blood of wild and OX40L$^{-/-}$ mice. (D) The proportion of CD25^{+}Foxp3^{+} Tregs in CD4^{+} T cells in the peripheral blood of wild and OX40L$^{-/-}$ mice. ** *P*<0.01 and *** *P*<0.001 represent a significant difference between wild group and OX40L$^{-/-}$ group. Data represent mean ± SD (unpaired t test was used).

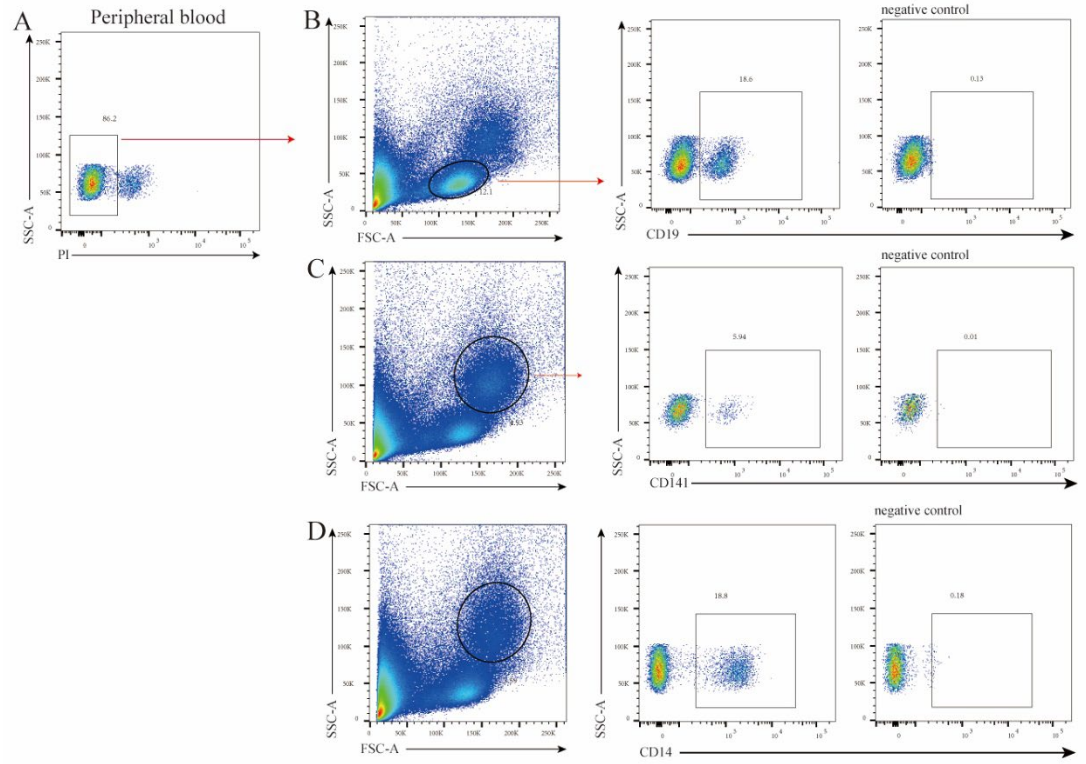

Figure S3. **The gating strategy and negative control for sorting peripheral blood CD19$^+$, CD141$^+$, or CD14$^+$ cells**. (A) The live/dead cell staining for peripheral blood single cell. (B) Gating strategy and negative control for sorting CD19$^+$ cells in peripheral blood. (C) Gating strategy and negative control for sorting CD141$^+$ cells in peripheral blood. (D) Gating strategy and negative control for sorting CD14$^+$ cells in peripheral blood.

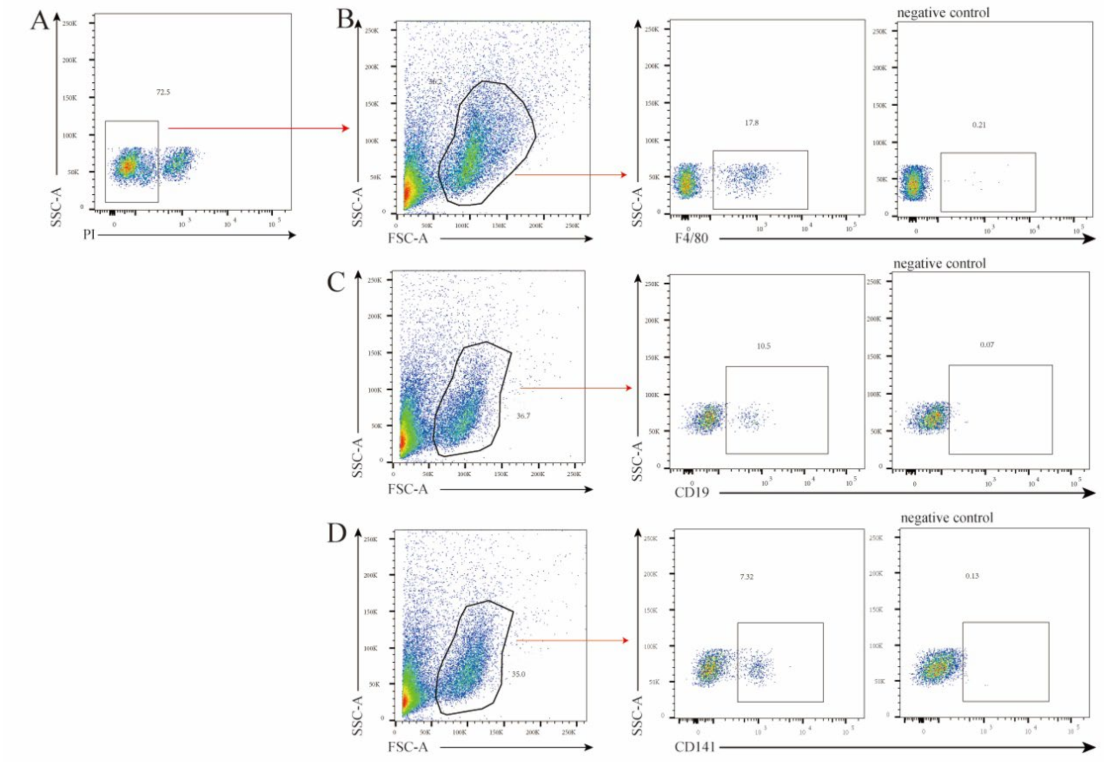

Figure S4. **The gating strategy and negative control for sorting synovial tissue CD19^{+}, CD141^{+}, or F4/80^{+} cells**. (A) The live/dead cell staining for synovial tissue single cell. (B) Gating strategy and negative control for sorting F4/80^{+} cells in synovial tissue. (C) Gating strategy and negative control for sorting CD19^{+} cells in synovial tissue. (D) Gating strategy and negative control for sorting CD141^{+} cells in synovial tissue.
